# A Procedure for Determining Tire-Road Friction Characteristics Using a Modification of the Magic Formula Based on Experimental Results

**DOI:** 10.3390/s18030896

**Published:** 2018-03-17

**Authors:** Juan A. Cabrera, Juan J. Castillo, Javier Pérez, Juan M. Velasco, Antonio J. Guerra, Pedro Hernández

**Affiliations:** 1 Mechanical Engineering Department, University of Malaga, 29071 Málaga, Spain; juancas@uma.es (J.J.C.); javierperez@uma.es (J.P.); juanmav@uma.es (J.M.V.); ajguerra@uma.es (A.J.G.); 2 Laboratory of the Murcia State Roads Demarcation, 30071 Murcia, Spain; pfhernandez@fomento.es

**Keywords:** friction coefficient measurement, tire model, skid resistance tester, tire parameters identification

## Abstract

Knowledge of tire-road friction characteristics is essential for the proper performance of most relevant vehicle active safety systems. Therefore, its determination is necessary to improve the effectiveness of these systems and to avoid or reduce the consequences of traffic accidents. For this reason, there is a great deal of literature concerning methods and devices for measuring and modeling tire-road friction. Most of these methods have focused on determining the road friction resistance, taking only road composition and making measurements in wet conditions into account. However, friction forces are also dependent on the tire type, since the contact is established between the tire and the road in real driving conditions. Thus, the type and characteristics of the tire have to be considered in the study of the interaction between the vehicle and the road. The aim of this work is to unify the study of the friction coefficient, taking into consideration the two existing bodies involved in the contact, i.e., the tire and road and the main factors that influence the forces in the contact. To this end, a modification of the Pacejka Magic Formula is proposed to include the effects of the main parameters that influence the contact, such as road composition and its state, tire type, vehicle speed, and slip between the tire and the road. To do so, real tests have been conducted on several roads and with different operating conditions. As a result, a more accurate tire-road friction model has been obtained.

## 1. Introduction

The main interaction between a vehicle and the physical environment is the contact between the tires and the road. The knowledge of friction characteristics of this contact is crucial for the improvement of vehicle active safety systems, as well as in the design of roads and infrastructures. A reduction of traffic accidents and the effects they cause can be achieved thanks to improvements in these two research lines. Therefore, the study of the dynamic interaction between vehicle and road is very important to enhance vehicle safety, but it can also be used to deal with other problems related to vehicles such as economy, noise emission, and transportation quality.

Due to the presence of two solids composed of different materials, the adherence conditions in the contact zone are affected by multiple factors. The most important factors that affect the friction characteristics are highlighted next:

- Composition, type, and tire condition (rubber type, tread, inflation pressure, temperature)
- Composition and type of road (macrotexture and microtexture)
- Existence of external agents (rain, ice, snow, dust, ...)
- Sliding speed in tire-road contact

Therefore, methods to measure and estimate the friction in the tire-road contact have attracted a great deal of attention and are still of the utmost importance for research groups of vehicular dynamics and for groups involved in the development of transport infrastructures. Nowadays, there are many proposed methods to measure the friction in tire-road contact. Some methods focus on the measurement of the road composition and its characteristics. Generally, these methods determine the road texture using two parameters: the macrotexture and the microtexture [1,2,3,4]. Subsequently, the texture is related to the friction characteristics of the surface.

The macrotexture depends on the size and shape of the mixture used in the asphalt composition. It has a big influence on the hysteresis component of friction and in the tire-road adherence when the vehicle is traveling at high speeds, especially with wet asphalt, since it improves water evacuation. The microtexture refers to the fine-scale texture of the road surface irregularities that interacts with the tire. It is mainly responsible for the generated friction force in the contact zone due to its contribution to the existence of local bonds between the tire rubber and the surface.

The macrotexture measurement is mainly carried out by means of two methods. The first one is the volumetric or sand patch method, in which a known quantity of sand or crystal spheres is spread evenly over the pavement making a circle. Next, the diameter of the circle is measured and the average texture depth is obtained, which is called ‘Mean Texture Depth‘ (MTD) (American Society for Testing and Materials (ASTM) E965). The second one is known as ‘Circular Track Meter’. It makes use of a high frequency laser to measure the profile of the road, obtaining the ‘Mean Profile Depth’ (MPD) (ASTM E2157). Microtexture can be measured by microscopy and stereography, although there are authors who describe other indirect methods to determine it [5].

The aforementioned methods do not measure the road friction resistance directly, but some factors that are related with it. On the other hand, there are some methods that measure the friction resistance under certain operation conditions. One of the most used is the ‘British Pendulum Tester’ (BPT) (ASTM E303), in which a pendulum with a rubber slider measures the energy loss when the rubber slider edge is propelled over a wet test surface. This loss of energy is related to the friction force between the rubber and the road. The measure obtained is called British Pendulum Number (BPN) or Skip Resistance Tester (SRT). It can be used to measure the longitudinal or the lateral friction coefficient, depending on the orientation of the pendulum. Another commonly used method is the ‘Sideway-force Coefficient Routine Investigation Machine’ (SCRIM), in which a truck-mounted machine is used to carry out continuous measurement of frictional force. The truck is equipped with a tank with water to wet the test road uniformly. It makes use of a fifth wheel with a slick tire that can rotate freely with a slip angle of 20°. The lateral force on the wheel measured by a load cell is divided by the vertical force used to calculate the sideway force coefficient value (SFC). A further method also based on the measurement of the lateral friction coefficient is the Mu-Meter (ASTM E670), in which a vehicle tows a trailer with two test wheels that rotate freely with an angle between them of 15°. As in the previous method, the road is wet, and the resulting lateral force acting between the test tires is measured with a load cell. The two previous friction resistance measurement methods use a freely rotating wheel, so the slip ratio between the tire and the road is null. There are also methods in which a relative slip between the tire and the road is forced or even locks the wheel completely. For example, the ‘Locked-Wheel Skid Trailer’ (ASTM E274) measures the Longitudinal Friction Coefficient on a wet surface when the slip is equal to one, that is, with the wheel fully locked.

The previously described methods, like others existing in literature, measure the friction resistance at different speeds. For example, the British pendulum measures friction at low speed (typically around 10–12 km/h), while the SCRIM and the Mu-Meter can perform the measurements at higher speeds, reaching up to 100 km/h or more. Furthermore, the values obtained by these methods are very different from each other, since some of them use a wheel that moves with a different slip angle or a different slip ratio. Others, like the pendulum, do not even measure with a tire, but with a rubber shoe. 

This casuistry of measurement methods and results has made some researchers consider the need to harmonize or standardize the measures obtained with each one of the devices used. For example, Schlösser [6] proposed a formula to obtain the longitudinal and lateral friction coefficients from the measurements of the British pendulum (SRT) and the texture (MTD). Leu and Henry [7] proposed the Penn State model, in which the measurement of the macro-texture (MTD) or (MPD) is related to the sliding speed. Likewise, Mancuso [8] suggested an alternative method to take into account the Stribeck effect, the macrotexture, and the depth of the water layer, in addition to the speed. Wambold et al. [9] proposed the Permanent International Association of Road Congresses (PIARC) model based on the measurements of the macrotexture made with various testing devices and created the International Friction Index (IFI) reported at a slip speed of 60 km/h.

Schlösser [6] also pointed out the importance of different factors in obtaining the friction resistance in his work, establishing an order of importance among them. According to this author, the factors that are most influential in the determination of the friction resistance are the characteristics of the road, the type of tire, and the speed at which the measurement is performed. All the methods proposed to obtain the friction resistance take road characteristics into consideration somehow. They also conduct the tests at different speeds. As mentioned before, the results can later be harmonized by the (IFI). However, none of them consider the influence of the different types of tires that a vehicle can equip.

There are many tire models in literature that take into account their characteristics to provide a friction resistance estimation. These models can be classified into physical and empirical models. Within the physical models, in which the behavior of the tire is modelled by differential equations, we could mention the Dugoff [10], Brush [11,12], LuGre [13], Gim [14], and Li [15] models. On the other hand, tests are carried out, and the measured data are used to fit the parameters of a pre-defined formula to reproduce tire behavior, yielding the so-called empirical models. Burckhardt [16] and Pacejka’s [17] proposals are well-known examples of empirical models. One of the most used models is the Pacejka Magic Formula [17], in which the coefficients of empirical equations are fitted to match the measured data. The equations can predict the friction forces on the tire with great precision. In this case, the inputs to the equations are the slip ratio, the slip angle, and a parameter that is related to the type of road in contact with the tire. The fitted parameters of the equations of the Magic Formula are specific to each tire, so tests have to be carried out previously to obtain them.

According to previous paragraphs, the most important factors in the estimation of the friction resistance can be integrated if the model of the Magic Formula and the measurements of macro-texture and microtexture made on the roads can be combined. The main drawback of the tire model proposed by Pacejka is that the influence of the contact surface is modelled by a single parameter, which makes the friction coefficient curves adjust linearly to the referred parameter. Furthermore, to estimate the friction resistance properly, we must take into account not only the macrotexture and microtexture but also the speed at which the measurements are made and the sliding speed. These parameters are not considered in the Magic Formula, even though they are significant in the obtaining of the friction coefficients.

As it has previously been stated, physical models [10,11,12,13,14,15] use analytical solutions of differential equations to estimate the force in the contact patch. These models can take into account some of the parameters previously mentioned such as vehicle speed and slip ratio. However, they also require the knowledge of some parameters that have to be obtained for each tire to be modelled. These parameters, such as patch length, longitudinal stiffness, and damping coefficients of the tire, among others, are difficult to obtain, requiring complicated tests to measure them in some cases.

Magic formula is considered a standard tire model in the industry and within scientific community for vehicle dynamics. It has a high level of correlation with tire test data and can be easily implemented in vehicle dynamics simulation programs. Moreover, it can be used in real time simulations thanks to its simplicity and low computational time compared to other tire models, i.e., it can provide accurate estimations by only evaluating simple mathematical expressions. Therefore, a modification of the Magic Formula to take into consideration the influence of the vehicle speed and the slip ratio in the road-tire friction modelling is of great interest. To this end, a three-parameter multiplication factor is added to the Magic Formula. The parameters are experimentally fitted to match the measured friction coefficient behavior when the slip is 1, that is, when the wheel is locked. This way, the longitudinal coefficient of friction will be obtained at any speed taking into account the characteristics of the tire and the road.

Tests have been conducted on different road types with a sensorized vehicle developed by this research group in order to test the effectiveness of the method. Measures were also carried out with the British pendulum and SCRIM. Once the model of the Modified Magic Formula was fitted, the corresponding values of the lateral friction coefficient were calculated for the velocities and test conditions of the British pendulum and the SCRIM, checking the correspondence of the results obtained.

The rest of the paper is organized as follows: First, the longitudinal and lateral force equations of the Magic Formula Tire Model are introduced in Section 2. The sensorized vehicle developed by this research group, which was used to conduct experimental tests, is described in Section 3. Next, Section 4 includes the experimental results obtained with the test vehicle and with the British Pendulum and the SCRIM on conventional roads. A discussion about the results obtained is carried out in Section 5. Finally, Section 6 draws the conclusions of this work.

## 2. Tire Model

The Pacejka Magical Formula is a standard method widely used in vehicle dynamics studies and throughout industry to describe the tire-road interaction at the contact patch [17]. This model is based on conducting real tests with the tires to fit the formula coefficients to the test data. After fitting coefficients of the equations, longitudinal and lateral forces can be estimated for pure and combined slip conditions. In this work, this tire model is used to obtain the friction coefficient between the tire and the road.

The Magic Formula at pure slip conditions is expressed as:

(1) y(x)=D·sin[C·atan{B·x−E·(B·x−atan(B·x))}]

with:

(2) $$\begin{array}{c} Y\left(x\right)=y\left(x\right)+S_{v}\\ x=X+S_{h} \end{array}$$

Equation (1) can reproduce the longitudinal or lateral forces or the auto aligning torque depending on the variable *X* used and the equation coefficients (*D*, *C*, *B*, *E*, *S_v_* and *S_h_*). Therefore, output variable *Y*(*x*) represents the braking and traction force *F_x_* when the input variable *X* is the slip ratio. *Y*(*x*) represents the lateral force *F_y_* or the auto aligning torque *M_z_* when the input variable *X* is the slip angle. In each case, specific coefficients have to be calculated from the parameters obtained in the tire tests. Depending on the force to be modelled, coefficients are calculated according to the following equations. Subindexes *x* and *y* have been used to differentiate between longitudinal and lateral equation parameters. Auto aligning torque is not used in this paper. Parameters descriptions are presented in Table 1:

Longitudinal force (*F_x_*):

(3) $$D_{x}=\mu_{x}\cdot F_{z}$$

(4) $$\mu_{x}=\left(P_{DX1}+P_{DX2}\cdot df_{z}\right)\cdot \lambda_{\mu x}$$

(5) $$df_{z}=\frac{F_{z}-F_{z0}}{F_{z0}}$$

(6) $$C_{x}=P_{CX1}$$

(7) $$B_{x}C_{x}D_{x}=F_{z}\cdot \left(P_{KX1}+P_{KX2}\cdot df_{z}\right)\cdot e^{-P_{KX3}\cdot df_{z}}$$

(8) $$B_{x}=\frac{B_{x}C_{x}D_{x}}{C_{x}D_{x}}$$

(9) $$E_{x}=\left(P_{EX1}+P_{EX2}\cdot df_{z}+P_{EX3}\cdot df_{z}^{2}\right)\cdot \left(1-P_{EX4}\cdot sign\left(s+S_{hx}\right)\right)$$

(10) $$S_{hx}=P_{HX1}+P_{HX2}\cdot df_{z}$$

(11) $$S_{vx}=\left(P_{VX1}+P_{VX2}\cdot df_{z}\right)\cdot F_{z}$$

Parameter $\lambda_{\mu x}$ is related to the type of road. Its value is within range [0–1]. Finally, *X = s* is the slip ratio, which is defined as:

(12) $$s=1-\frac{\omega \cdot R}{v_{x}}$$

in which *ω* is the angular velocity of the tire, *R* the effective radius of the tire, and $v_{x}$ the vehicle longitudinal speed.

Lateral force (*F_y_*):

(13) $$D_{y}=\mu_{y}\cdot F_{z}$$

(14) $$\mu_{y}=\left(P_{DY1}+P_{DY2}\cdot df_{z}\right)\cdot \left(1-P_{DY3}\cdot \gamma^{2}\right)\cdot \lambda_{\mu y}$$

(15) $$C_{y}=P_{CY1}$$

(16) $$B_{y}C_{y}D_{y}=F_{z0}\cdot P_{KY1}\cdot sin\left(2\cdot atan\left(\frac{F_{z}}{P_{KY2}\cdot F_{z0}}\right)\right)\cdot \left(1-P_{KY3}\cdot \left|\gamma \right|\right)$$

(17) $$B_{y}=\frac{B_{y}C_{y}D_{y}}{C_{y}D_{y}}$$

(18) $$E_{y}=\left(P_{EY1}+P_{EY2}\cdot df_{z}\right)\cdot \left(1-\left(P_{EY3}+P_{EY4}\cdot \gamma \right)\cdot sign\left(\alpha +S_{hy}\right)\right)$$

(19) $$S_{hy}=P_{HY1}+P_{HY2}\cdot df_{z}$$

(20) $$S_{vy}=\left(P_{VY1}+P_{VY2}\cdot df_{z}\right)\cdot F_{z}$$

in which *X* = α is the slip angle and γ is the camber angle.

## 3. Test Vehicle

**Figure 1:**
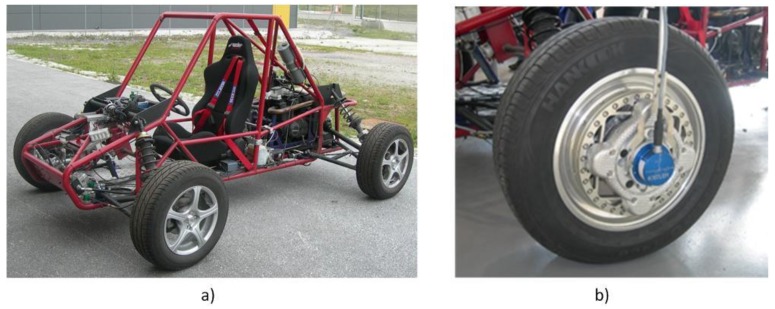
(**a**) IMMa sensorized vehicle. (**b**) Detail of the measuring rim.

A sensorized vehicle, designed by this research group, was used (Figure 1a) to conduct real tests on conventional roads. The vehicle was equipped with sensors to measure the variables involved in braking processes, including an inertial unit and a high frequency Global Positioning System (GPS) system. A dynamometric rim was also installed on the vehicle to provide longitudinal, lateral, and vertical tire forces, as well as self-aligning moment measurements (Figure 1b).

**Figure 2:**
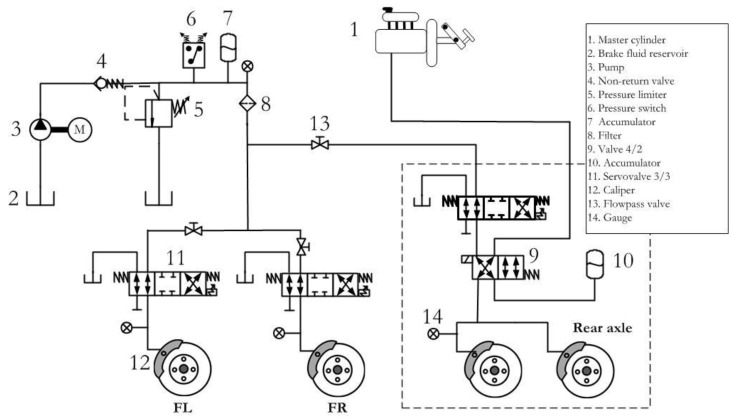
Brake-by-Wire braking system hydraulic diagram.

The advantage of using this vehicle is that the braking tests can be scheduled and conducted in real driving conditions. The brake pressure in each one of the brake circuits of the vehicle is independently controlled with a real time system by means of fast-response servo-valves (Figure 2). The system works as follows in non-automatic mode: valve 2/4 (9) remains in an idle position when no voltage is applied to it. This way, pressure at the brake pump (1) is transmitted to the three braking circuits through the 4-way 2-position valve (9) and the servovalves (11). When working in this configuration, the brake system operation is similar to a traditional system installed in a vehicle without Antilock Braking System (ABS). On the other hand, in automatic mode, the pressure generated by the pump (3) is transmitted to the braking circuits through the 4/2 valve (9), which is energized in this mode. The servo valves (11) regulate the amount of pressure applied to each brake piston (12) independently and according to the command signal sent by the control system. In this situation, the user is acting on the braking pedal but has no direct control of the braking process. Consequently, the brake system works like a Brake-by-Wire system under these operating conditions.

The vehicle is equipped with Hankook (Seul, South Korea) 205/65 R15 tires. These tires were previously tested and the parameters of the Pacejka model were obtained [18,19]. Table 2 shows the values of the Pacejka parameters for longitudinal and lateral force of this tire.

Measurements made with the British pendulum method and a SCRIM survey vehicle on the test roads were provided by the Laboratory of the Murcia State Roads Demarcation.

## 4. Test and Results

**Figure 3:**
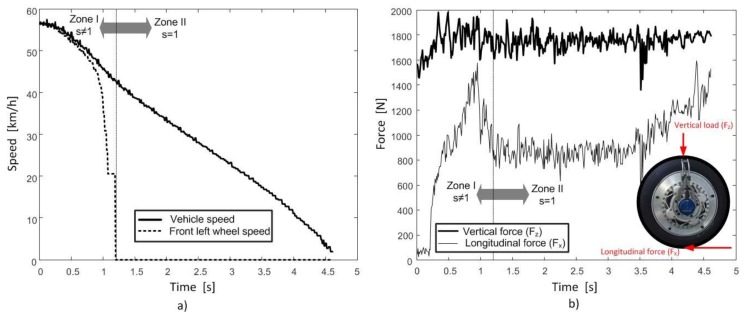
Example of data measured in a regular test. (**a**) Vehicle and wheel peripheral speed. (**b**) Tyre forces.

Several tests were carried out on different roads. The following guidelines were established for all tests:

- Tests were conducted in a straight line and with the road in two different conditions: wet and dry. A minimum of two tests were conducted in each surface.
- Manufacturer’s recommended inflation pressure was fitted in all tires (2 bars).
- The test vehicle was first accelerated up to a predefined speed. The front wheel brake pressures were increased when the speed was reached. No ABS was used, so the wheels locked during the tests.
- The following data were recorded: longitudinal and vertical forces on the front left wheel and angular velocities of all the wheels. As an example, Figure 3 shows the measured data in a regular test. There are two differentiated zones in Figure 3, one with slips s ≠ 1, in which the wheel is not locked, and another one with s = 1, in which the wheel is locked. Only the test data from zone II (s = 1) are used to obtain the new parameters of the model of the Modified Magical Formula. The data of zone I (s ≠ 1) will be used to compare the model with the experimental data.
- The rear wheels were not braked. Therefore, the linear speed of the wheel was taken as the longitudinal speed of the vehicle.
- A Rotating Wheel Dynamometer model P625 by Kistler ® (Winterthur, Switzerland) installed in the front left wheel was used to measure vertical and longitudinal tire forces.

Slip, *s*, was calculated using the Equation (12), and the friction coefficient was calculated by means of the data provided by the dynamometric rim using the following equation:

(21) $$µ=\frac{F_{x}}{F_{z}}$$

in which µ is the friction coefficient, *F_x_* is the vertical load, and *F_z_* is the longitudinal force.

**Figure 4:**
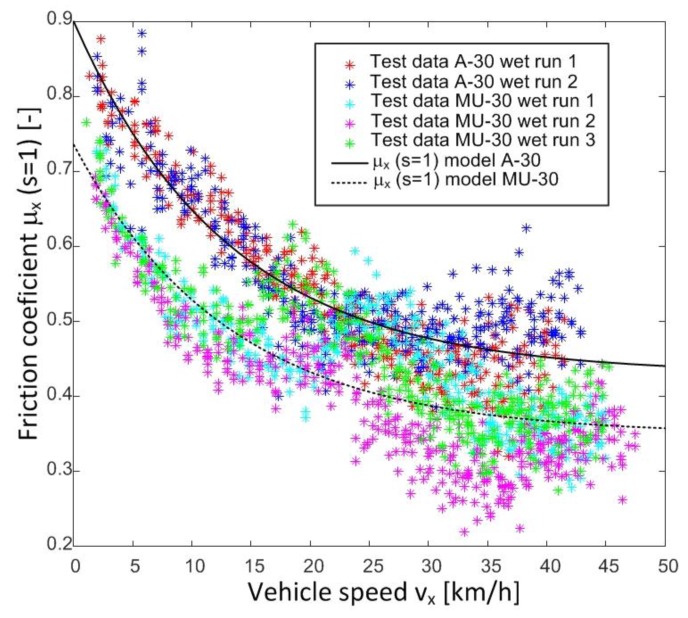
Friction coefficient vs vehicle speed. A-30 and MU-30 roads. Wet asphalt.

Figure 4 shows the data obtained in tests in two sections of the following roads of the Spanish national network: OP14 A-30 km. 84 (GPS Coordinates: 38.3539049935706 −1.51064523471407) and OP4 MU-30 El Palmar km. 0 (GPS coordinates: 37.9464322964378 −1.14911171797529). Figure 4 includes the results obtained in tests 2 and 3 conducted on A-30 and MU-30 test roads, respectively. All tests were carried out in wet conditions. As it can be observed, the longitudinal friction coefficient decreases progressively when the tire is locked (*s* = 1) and the vehicle speed increases [20]. This behaviour is not contemplated in parameter $\lambda_{\mu x}$ of the Magic Formula tire model, which only establishes the road type ($\lambda_{\mu x}$) with a constant value between zero and one. There are works that propose a model that reflects this aforementioned behaviour [7,8,9], but they relate the macrotexture and the speed. In our work, a model that can be adjusted by test data is proposed. This model modifies the value of parameter $\lambda_{\mu x}$ in the Pacejka tire model, and thus the tire-road contact behaviour observed in real tests can be reflected. In order to do this, the model will include the influence of microtexture and macrotexture of the road, vehicle speed, and the slip coefficient between the tire and the road.

The proposed model is shown in the following equation:

(22) $$\lambda_{\mu x}=PLX1+PLX2\cdot e^{-PLX3\cdot s\cdot v_{x}}$$

in which *PLX*1, *PLX*2 y *PLX*3 are the new parameters that will be fitted to the test data with an optimization process based on genetic algorithms [19], *s* is the slip ratio as defined in Equation (12), and $v_{x}$ is the vehicle linear speed. This way, parameter $\lambda_{\mu x}$ is still related to the type of road and its adhesion characteristics, but it can also include the effects of speed and other factors in the friction coefficient.

Some transition can be observed in some tests at speeds around 25 km/h (see Figure 4). This transition can be due to the Stribeck effect or to the surface characteristic. In the first case, this effect has already been described by some authors [21,22]. However, this transition is not modelled in our approach, since it has not been detected on all surfaces. New tests on different surfaces are required to study what surfaces and test conditions this transition is detected. In such cases, new parameters will be added to the modification proposal of Pacejka’s formula to model this behaviour.

Table 3 includes the optimized parameter values for the wet asphalt test on roads A-30 and MU-30. Data of two tests on each surface were used during the optimization process. The mean of the values obtained for the two tests was considered as the optimized parameters. As mentioned before, the basic data for the optimization process are the friction coefficient and vehicle speed. Therefore, other test methodologies, such as the Locked-Wheel Skid Trailer and Delft-Tire Test Trailer, can be used provided they can record this required data.

**Figure 5:**
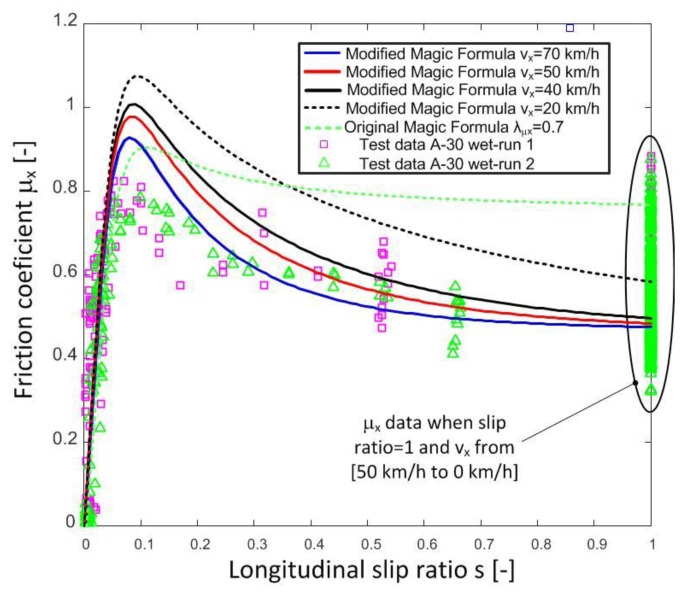
Longitudinal friction coefficient versus slip with different speeds. Highway A-30. Modified model of the Magic Formula.

**Figure 6:**
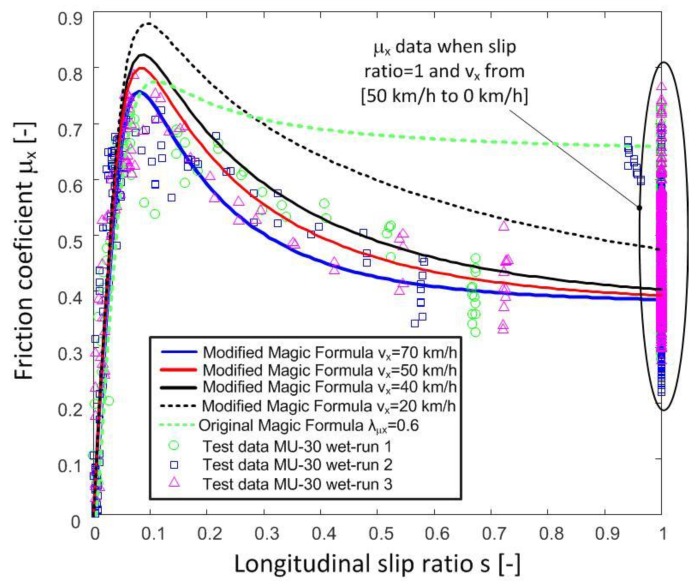
Longitudinal friction coefficient versus slip with different speeds. Highway MU-30. Modified model of the Magic Formula.

Now, the longitudinal friction coefficient vs the longitudinal slip ratio with the optimized values can be plotted as a function of the longitudinal speed. Figure 5 shows the test data obtained on road A-30 and the friction coefficient provided by the proposed modification of the Magic Formula tire model at different longitudinal speeds. Similarly, Figure 6 includes the results obtained on road MU-30.

It can be observed that the proposed model fits the measured test data adequately on both roads. In the first case, the estimated values match the measures reasonably well, but in the highest friction coefficient area, a higher error is observed. On the second road, estimates are adequate for all longitudinal slip ratios. On the other hand, it can be observed that the original Pacejka model with a constant λµ_x_ value does not reproduce the friction coefficient properly when the slip ratio is greater than 0.1. Higher reduction of the friction coefficient vs. slip ratio than the one predicted by the unmodified Magic formula is measured in both tests.

**Figure 7:**
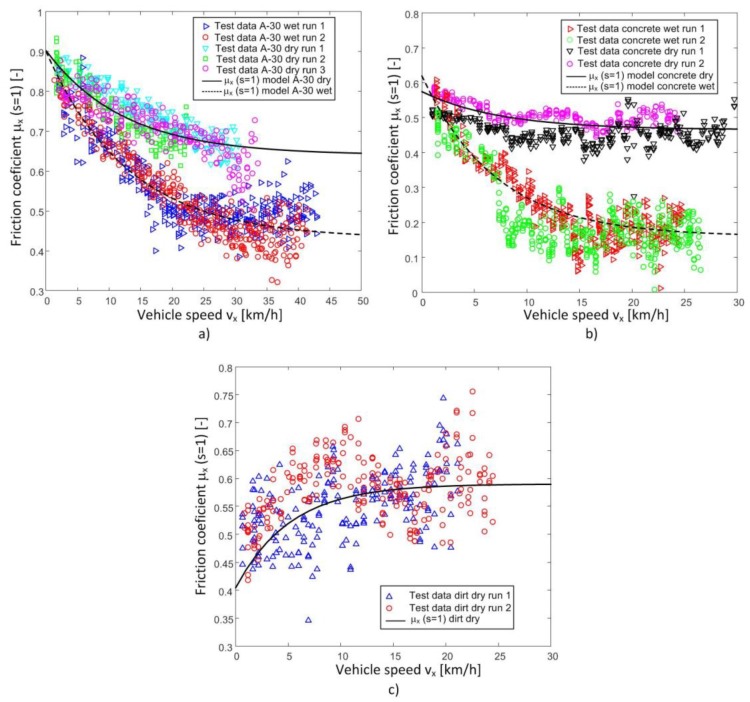
Friction coefficient versus longitudinal speed of the vehicle in different road types. (**a**) Wet and dry A-30 road. (**b**) Wet and dry concrete road (**c**) Dry dirt road.

Similar tests, in which the wheel is locked and the tire forces and vehicle speed are measured, were also made on different road conditions: dry asphalt, dry concrete, wet concrete, and dirt road. Figure 7 shows the friction coefficients vs the vehicle speed test data series obtained with these surface conditions.

As it can be seen in Figure 7, the decrease in the friction coefficient when the speed increases is less pronounced when the road is in dry conditions. It can also be observed that the effect is the opposite on the dirt road, that is, the friction coefficient increases with speed. This fact is due to the accumulation of soil when the wheel is locked. This accumulation is greater at higher speed, which produces a higher friction coefficient at higher speeds when the wheel is locked. A similar effect is also discussed in literature when braking on snow covered roads.

Table 4 shows the values of parameters $\lambda_{\mu x}$ of the modified tire-road friction model for 4 road conditions in which real tests were also conducted.

**Figure 8:**
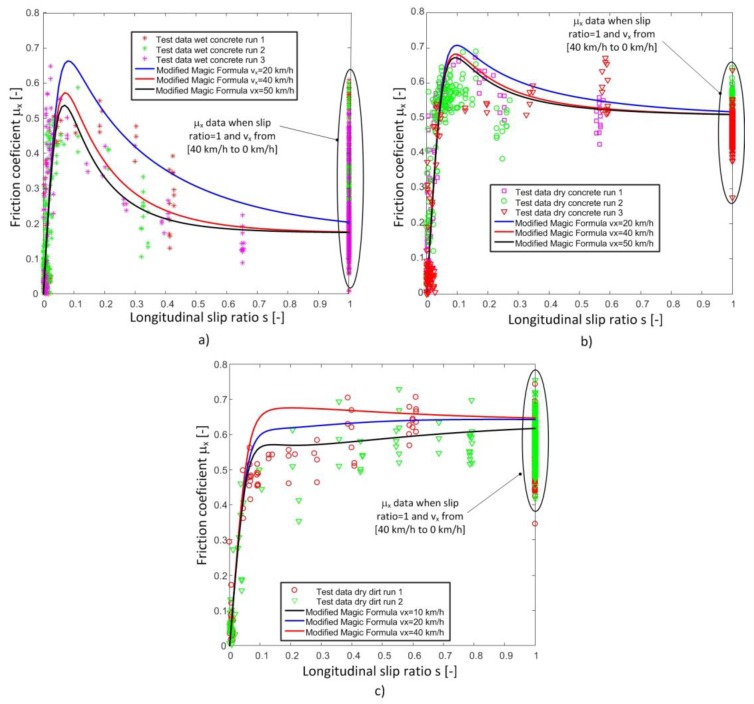
Friction coefficient versus slip at different speeds. (**a**) Wet concrete road. (**b**) Dry concrete road. (**c**) Dry dirt road.

The longitudinal friction coefficients estimated with the proposed modification of the Magic Formula are also shown in Figure 8. As it can be seen, the estimated values closely match the measured data in all tests in which the modified Magic Formula is evaluated at speed values within the speed range in which the tests are conducted.

### 4.1. Comparasion of Conventional Friction Measurement Methods

Once the tests on the proposed road types have been described and the modified model of the Magic Formula has been introduced, a comparison with the friction resistance measurements carried out with the British Pendulum (Munro Instrument, Essex, UK) and the SCRIM is made on test roads A-30 and MU-30.

The Laboratory of the Murcia State Roads Demarcation carried out the measurements with the aforementioned devices on the road sections that have been described. Obtained results shown in Table 5 also include the measurements made with the volumetric or sand patch method (MTD) and with the Circular Track Meter (MPD).

This comparative is intended to evaluate the difference between the measurements carried out with the conventional methods and the expected values to be obtained with a real tire in similar conditions. Therefore, it is necessary to know the measurement conditions of these devices to simulate these measurements with our tire-road friction model. These conditions are included in Table 6. As it was stated in the introduction section, test conditions and measured values with these methods are quite different. For this reason, criteria have been established to harmonize the measures [9].

**Figure 9:**
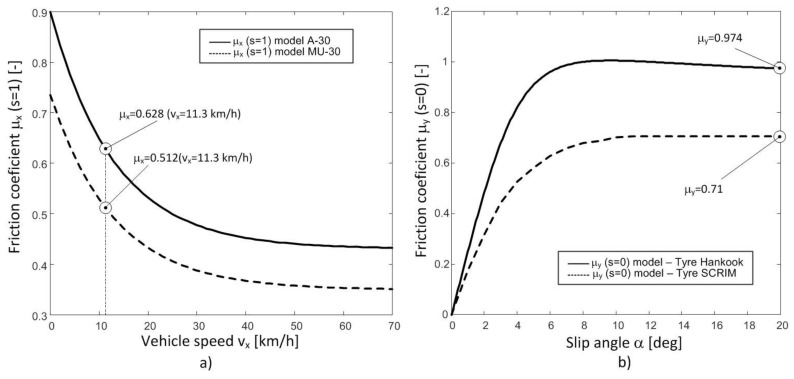
Longitudinal and lateral friction coefficients. (**a**) Longitudinal friction coefficient versus vehicle speed on roads A-30 and MU-30. (**b**) Lateral friction coefficient. Hankook and SCRIM tires on steel surface.

The proposed modified model provides the longitudinal friction coefficient as a function of the slip ratio with null slip angle. The British Pendulum is used to estimate the longitudinal friction using a rubber slider on a wet road. Figure 9a shows the model of the longitudinal friction coefficient in the two sections of road A-30 and MU-30 as a function of the longitudinal speed. The expected friction coefficient coincides with the friction coefficient provided by the model at the same test speed of British Pendulum, 11.3 km/h.

In the case of the SCRIM, the tire used is different from the Hankook 205/65R15, so the parameters of the lateral model of this tire cannot be used (Table 2). Besides, the slip angle is not null. Therefore, it is necessary to harmonize the measurements with the SCRIM tire to be able to estimate the expected results with the *Hankook* tire. Domenichini et al. [23] obtained the parameters of the Magic Formula for the tire used in the *SCRIM*. Figure 9b shows the lateral friction coefficient curves of both tires. The Hankook tire provides higher values of the lateral coefficient than the *SCRIM* tire. A similar behavior is reported in [23,24]. Furthermore, according to [2], a multiplying factor can be determined from these two curves to harmonize the two tires by means of the friction coefficient value for a slip angle of 20°. In this case, this multiplying factor is the following:

(23) $$LHS=\frac{\mu_{y}{\left(\alpha =20^{0}\right)}_{HANKOOK}}{\mu_{y}{\left(\alpha =20^{0}\right)}_{SCRIM}}=1.37$$

Therefore, the value measured by the *SCRIM* is multiplied by the calculated *LHS* parameter to obtain the value of the *SCRIM* expected to be provided by the used tire on road A-30 and MU-30 with *SCRIM* test conditions. As a summary, all the expected values with the *Hankook* tire are presented in Table 7.

### 4.2. Braking Distance

A relevant parameter in infrastructures design and in the setting of speed limits is the braking distance. This distance is calculated in the worst scenario, that is, when the wheels of the vehicle are locked, which yields lower values of the longitudinal friction coefficient. The following differential equation can be used to estimate the braking distance:

(24) $$m\cdot \frac{dv}{dt}=\mu_{x}\cdot F_{z}$$

in which *m* is the vehicle mass, *v* is the linear velocity, $\mu_{x}$ the longitudinal friction coefficient when *s* = 1, and $F_{z}$ is the vertical force. As all the wheels of the vehicle are locked, the vertical force can be obtained from $F_{z}=m\cdot g,$ and coefficient can be obtained $\mu_{x}$ from Equation (22) for *s* = 1. Therefore, the following relationship can be obtained:

(25) $$\frac{dv}{dt}=\left(PLX1+PLX2\cdot e^{-PLX3\cdot v}\right)\cdot g$$

The vehicle speed when the wheels are locked can be obtained by integrating Equation (25):

(26) $$v=\frac{1}{PLX3}\cdot ln\left(k_{1}\cdot e^{k_{2}\cdot t}-\frac{PLX2}{PLX3}\right)$$

in which:

(27) $$k_{1}=e^{PLX3\cdot v_{0}}+\frac{PLX2}{PLX3}$$

(28) $$k_{2}=3.6\cdot PLX1\cdot PLX3\cdot g$$

Finally, the braking distance can be estimated by integrating Equation (26), between time *t* = 0 and *t* = *tf*, which is the moment when vehicle speed is zero.

(29) $$d_{brake}=\frac{1}{3.6\cdot PLX3}\int_{0}^{tf}ln\left(k_{1}\cdot e^{k_{2}\cdot t}-\frac{PLX2}{PLX1}\right)\cdot dt$$

in which:

(30) $$tf=\frac{1}{k_{2}}\cdot ln\left(\frac{1+\frac{PLX2}{PLX1}}{k_{1}}\right)$$

Table 8 shows the braking distance obtained with Equation (29) on roads A-30 and MU-30 with different initial speeds.

## 5. Discussion

In this work, the two main elements involved in obtaining the friction coefficient, that is, the tire and the road, are taken into account. For this reason, a proposal of modification of the Magic Formula tire model in the case of longitudinal force (see Equation (22)) based on the results obtained in experimental tests is introduced. The variation of the Magic Formula model has been carried out with the inclusion of three new parameters (PLX1, PLX2 y PLX3), which have to be fitted by carrying out tests in real conditions. This modification allows adapting the tire model to any type of road and condition, thus, including the two determining factors in obtaining friction characteristics.

It has been proved that the following parameters are of great influence on the longitudinal friction coefficient when the wheel is locked (*s* = 1): vehicle linear speed ($v_{x}$), road conditions (dry, wet), and road type (asphalt, concrete, unpaved). Parameter PLX1 reflects the influence of the microtexture in the road, while parameters PLX2 and PLX3 are related to the macrotexture and the water evacuation capacity, respectively. Therefore, parameter PLX3 is constant when the road type is the same and changes if the road type does as well (see Table 3 and Table 4). Also, note that the effect of decreasing the coefficient of longitudinal friction with the increase in speed is reversed in the case of the dirt road (see Figure 7). This can be attributed to the accumulation of soil in the locked wheel, which increases friction resistance.

The modification of the equation of the longitudinal friction coefficient of the Pacejka Magic Formula by means of the proposed three new parameters fits the test data adequately. Besides, it provides better estimates of the friction coefficient vs slip ratio (Figure 5, Figure 6 and Figure 8). Therefore, the tire-road interaction has been characterized more realistically. It is well-known that the longitudinal force vs slip ratio curves are of great importance in the design of active safety systems such as ABS, Traction Control System (TCS), etc. On one hand, it allows knowing the maximum available force to brake or accelerate a vehicle. On the other hand, it can be used to determine the loss of effectiveness in braking and traction processes when a wheel is locked. The longitudinal coefficient friction when the wheels are locked $\mu_{x}$(s = 1) and maximum friction coefficient $\mu_{x}^{max}$ can be estimated more precisely with this new proposal than with the conventional Magic Formula (see Figure 4, Figure 5, Figure 6, Figure 7 and Figure 8).

Furthermore, it has been verified that friction measurements made with the British Pendulum and the SCRIM (widely used to measure friction resistance on roads) are lower than those obtained in our tests (see Table 7). This fact has also been reported in other works such as in [23,24]. Thus, it can indeed be derived that the influence of the tire on the friction resistance measure is very important and should be taken into account in the design of new roads. Besides, the braking distance of a vehicle with locked wheel has been estimated. To do so, the fact that the longitudinal coefficient friction increases when the speed of the vehicle decreases with locked wheel $\mu_{x}$(s = 1) has been taken into account, as it was observed in the real tests.

Finally, note that the study conducted in this work has focused on longitudinal behavior in the tire-road interaction. Similar behavior is also expected in the lateral model. To prove it, it is necessary to carry out tests with different slip angles, a different slip ratio, on different roads, and in different conditions.

## 6. Conclusions

This paper has presented a proposal for the modification of the Pacejka Magic Formula to model the tire-road contact forces more precisely than with the conventional approach. It has been shown that friction resistance measurements taken by standard devices do not use commercial tires like those equipped by the vehicles. It is, therefore, necessary to evaluate tire influence and include it in the models to determine friction resistance adequately.

On the contrary, the equation to model the longitudinal friction coefficient of the Magic Formula does not adequately contemplate the road type and road condition. This will affect the estimation of the maximum friction coefficient and the friction coefficient when the wheel is locked. This lack of accuracy has consequences in the design and functioning of vehicle active safety systems and in the estimation of braking distances.

Models of the longitudinal friction coefficient when the wheels are locked have to take into account vehicle speed, road type, and the conditions of the same. Standard methods only measure friction resistance in wet conditions. However, the friction coefficient has different kinds of behavior under different operating conditions.

In this paper, we propose a simple modification of the Magic Formula to take into account the influence of the speed in the friction coefficient. Tests have been conducted on different surfaces to evaluate the improvements on the friction coefficient modelling of the Magic formula with this modification. It has been observed that the friction coefficient estimation is more accurate when the modified formula is used. Further tests will be carried out on different roads and surface conditions to the extent of the results outlined in this work.

Finally, it has been verified that the values of friction resistance determined by the British Pendulum and the SCRIM on the tested roads are lower than those obtained in this work. Therefore, it is emphasized that the tire is an important factor to consider in the measuring of the friction resistance. This fact should be considered in the design of roads, speed limits, etc.

## Figures and Tables

**Table 1:** Pacejka’s Magical Formula coefficient description.

Longitudinal Coefficients	Lateral Coefficients
PCX1: C_Fx_ shape factor for longitudinal force	PCY1: C_FY_ shape factor for lateral force
PDX1: µ_x_ longitudinal friction at F_znom_	PDY1: µ_y_ lateral friction
PDX2: µ_x_ friction variation with load	PDY2: µ_y_ friction variation with load
PEX1: E_Fx_ longitudinal curvature at F_znom_	PDY3: µ_y_ friction variation with square camber
PEX2: E_Fx_ curvature variation with load	PEY1: Lateral E_FY_ curvature at F_znorm_
PEX3: E_Fx_ curvature variation with squared load	PEY2: E_FY_ curvature variation with load
PEX4: Factor in *E_Fx_* curvature while driving	PEY3: Zero order camber dependency of E_FY_ curvature
PKX1: *K_Fx_/F_z_* longitudinal slip stiffness at F_znom_	PEY4: E_FY_ curvature variation with camber
PKX2: *K_Fx_/F_z_* slip stiffness variation with load	PKY1: K_FY_/F_znom_ stiffness maximum value
PKX3: Exponent in *K_Fx_/F_z_* slip stiffness with load	PKY2: Load at which K_FY_/F_znom_ reaches maximum value
PHX1: *S_hx_* horizontal shift at F_znom_	PKY3: K_FY_/F_znom_ variation with camber
PHX2: *S_hx_* shift variation with load	PHY1: S_hy_ horizontal shift at F_znom_
PVX1: *S_vx_/F_z_* vertical shift at F_znom_	PHY2: S_hy_ shift variation with load
PVX2: *S_vx_/F_z_* shift variation with load	PVY1: S_vy_/F_z_ vertical shift at F_znom_
	PVY2: S_vy_/F_z_ shift variation with load

**Table 2:** Parameters for pure longitudinal and lateral force [19]. Tire 205/65R15.

Longitudinal Coefficient	Value	Lateral Coefficient	Value
PCX1	1.39708965	PCY1	1.276760
PDX1	1.10206790	PDY1	0.932775
PDX2	−0.18524061	PDY2	−0.128085
PEX1	−0.45925516	PDY3	1.019803
PEX2	−1.49950140	PEY1	−1.399340
PEX3	−2.46964541	PEY2	−0.074863
PEX4	−0.90674124	PEY3	0.178860
PKX1	38.50310903	PEY4	−8.252847
PKX2	2.03196267	PKY1	−17.36182
PKX3	−0.59108577	PKY2	2.293896
PHX1	−0.00227143	PKY3	−0.110362
PHX2	0.00193554	PHY1	0.001696
PVX1	0.05759227	PHY2	0.003882
PVX2	−0.02874956	PVY1	0.006931
		PVY2	0.018685

**Table 3:** Model parameters of the Modified Magic Formula tire.

λµx Coefficients	A-30	MU-30
PLX1: microtexture longitudinal friction [-]	0.430688	0.349478
PLX2: macrotexture longitudinal frictio [-]	0.469080	0.386194
PLX3: macrotexture shape factor [h/km]	0.076649	0.076649

**Table 4:** Tire model parameters of the modified Magic Formula. Dry asphalt, dry concrete, wet concrete, and dry unpaved surface.

λµ_x_ Coefficients	A-30 Dry	Concrete-Dry	Concrete-Wet	Unpaved-Dry
PLX1: microtexture longitudinal friction [-]	0.640353	0.465652	0.159353	0.590189
PLX2: macrotexture longitudinal friction [-]	0.261665	0.109246	0.460453	−0.185632
PLX3: macrotexture shape factor [h/km]	0.080955	0.134845	0.141727	0.192696

**Table 5:** Measures with British Pendulum and SCRIM on A-30 and MU-30.

Road	British Pendulum	SCRIM	MTD	MPD
OP14 A-30 84 + 600	57	42.71	2.90	1.63
OP4 MU-30 El Palmar 0 + 800	37	27.78	0.50	0.50

**Table 6:** Test conditions in the devices to measure the friction resistance.

Test Requirements	British Pendulum	SCRIM
Test speed	11.3 km/h	50 km/h
Test slip angle	0^0^	20^0^
Test slip ratio	S = 1	S = 0
Road condition	wet	wet
Friction element	Rubber slider	Flat tire

**Table 7:** Hankook parameter values in the sections A-30 and MU-30.

Road	British Pendulum/Hankook	SCRIM/Hankook
OP14 A-30 84 + 600	57/62.8	42.71/58.51
OP4 MU-30 El Palmar 0 + 800	37/51.2	27.78/38.06

**Table 8:** Braking distance at different initial speeds.

Road	Braking Distance	
OP14 A-30 84 + 600	(v_0_ = 50 km/h)	20.36 [m]
	(v_0_ = 100 km/h)	88.44 [m]
	(v_0_ = 120 km/h)	128.58 [m]
OP4 MU-30 El Palmar 0 + 800	(v_0_ = 50 km/h)	25.07 [m]
	(v_0_ = 100 km/h)	108.98 [m]
	(v_0_ = 120 km/h)	158.46 [m]
